# Primary grade 2 neuroendocrine tumor of the ileal mesentery: a case report

**DOI:** 10.1186/s40792-022-01482-x

**Published:** 2022-08-01

**Authors:** Shigemi Morishita, Shinichi Yoshida, Yasufumi Kamatani, Shinya Suzuhigashi, Masaki Kitou, Takuma Nasu

**Affiliations:** 1 Department of Surgery, Kagoshima Seikyou General Hospital, 5-20-10 Taniyama-chuou, Kagoshima City, Kagoshima 891-0141 Japan; 2 Department of Pathology, Kagoshima Seikyou General Hospital, 5-20-10 Taniyama-chuou, Kagoshima City, Kagoshima 891-0141 Japan

**Keywords:** Neuroendocrine tumor G2, Mesenteric tumor, Ileum

## Abstract

**Background:**

The prevalence and incidence of neuroendocrine tumors (NETs) are increasing worldwide. Primary mesenteric NETs are extremely rare. Solid tumors that arise in the mesentery are typically metastatic. We present an extremely rare case of a primary grade 2 NET (NET G2) in the ileal mesentery.

**Case presentation:**

A 54-year-old man was referred to our hospital for further examination of a previously diagnosed right mesenteric tumor. Mild tenderness was noted on the right side of the abdomen, but there were no palpable masses. Fluorodeoxyglucose-positron emission tomography (FDG-PET) revealed slight FDG uptake (maximum standardized uptake value, 2.0) in the right abdomen, and a benign or low-grade malignant tumor was suspected. We extracted the ileal mesenteric tumor with an ileal resection (90 cm). The cut surface of the 55 × 33 × 33 mm^3^ tumor was pale yellowish-white. Immunohistochemistry revealed diffuse staining for synaptophysin and chromogranin A, and focal staining for CD56. The Ki-67 index was 3%. The final pathological diagnosis was NET G2. The patient’s postoperative course was uneventful, and he developed no recurrence 1.5 years after surgery. Postoperative antitumor therapy was not performed for this patient because the histological diagnosis was NET G2, and it was determined that the tumor could be completely resected by surgery.

**Conclusions:**

We report an extremely rare case of primary ileal mesenteric NET. Mesenteric tumors that show slight FDG uptake on FDG-PET examination should be considered well-differentiated NET.

## Background

Neuroendocrine neoplasm (NEN) is a rare disease with a gradually increasing prevalence [1]. NENs are divided into well-differentiated neuroendocrine tumors (NETs) and poorly differentiated neuroendocrine carcinomas (NECs), based on the degree of tissue differentiation. Furthermore, according to the World Health Organization (WHO) classification 2019, it is classified as NET G1, G2, G3, and NEC G3 based on Ki-67, which is a proliferation marker that reflects prognosis (Table 1). We present an extremely rare case of a primary grade 2 NET (NET G2) in the ileal mesentery that demonstrated benign or low-grade malignant characteristics on preoperative fluorodeoxyglucose-positron emission tomography (FDG-PET).

**Table 1:** Incidence of NEN in Japan in 2016 [3]

Primary tumor site	Diagnosed number in 2016	Adjusted incidence/100,000 people in 2016
**Pancreas**	**1136**	**0.697**
**GI-NEN**	**5399**	**2.835**
Esophagus	262	0.098
Stomach	1042	0.482
Duodenum	442	0.195
Jejunum/ileum	97	0.046
Appendix	105	0.074
Colon	278	0.118
Rectum	3173	1.835
**All cases**	**6735**	**3.532**

*GI* gastrointestinal

## Case presentation

**Fig. 1:**
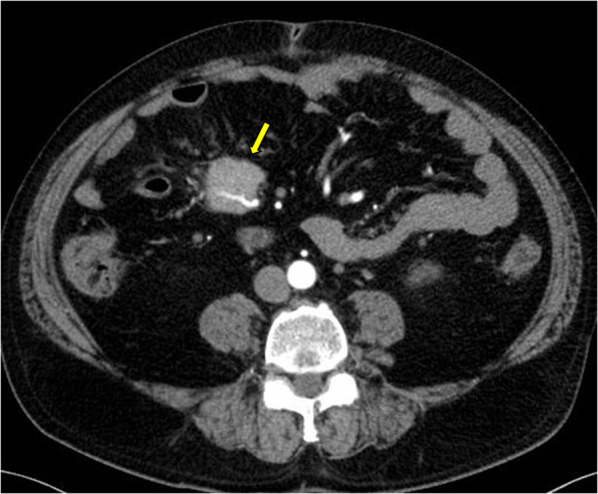
Contrast-enhanced computed tomography of the abdomen reveals a mesenteric lobulated mass with an artery running through the center (arrow)

**Fig. 2:**
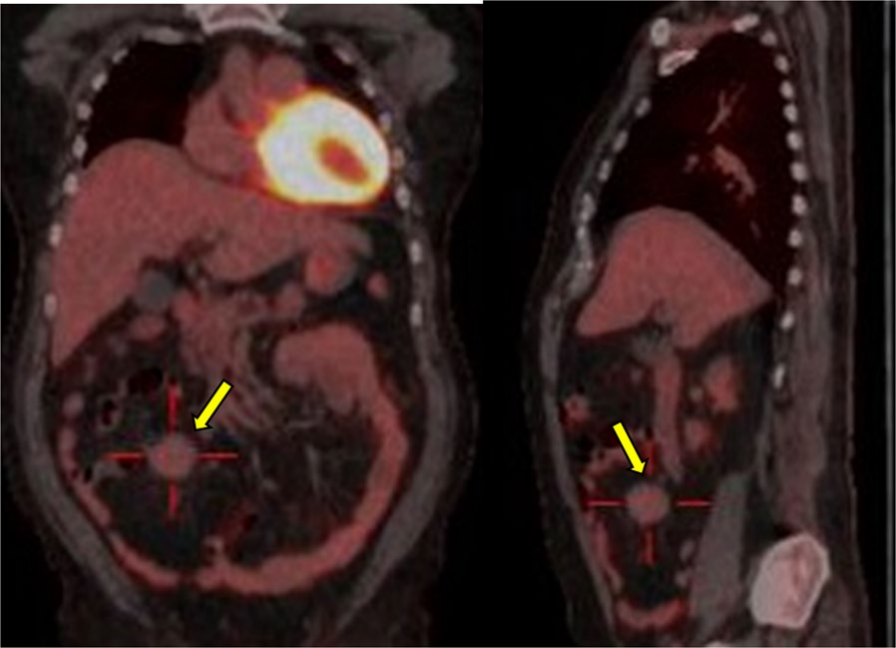
Slight fluorodeoxyglucose uptake (maximum standardized uptake value, 2.0) in the right abdomen (arrows). A benign or low-grade malignant tumor was suspected

A 54-year-old man was referred to our hospital after his previous doctor thoroughly examined him for right-sided abdominal pain. His previous diagnosis based on computed tomography (CT) was a right mesenteric tumor. Mild tenderness was noted on the right side of the abdomen, but there were no palpable masses. His medical history included diabetes mellitus and hypertension. Blood chemistry tests showed mild elevation of liver enzymes levels and elevated blood glucose and hemoglobin A1c levels, which are associated with diabetes mellitus. The tumor marker carcinoembryonic antigen level was mildly elevated at 6.2 ng/mL. Contrast-enhanced abdominal CT revealed a mesenteric lobulated mass with an artery running through the center (Fig. 1). FDG-PET revealed slight FDG uptake (maximum standardized uptake value, 2.0) in the right abdomen; hence, a benign or low-grade malignant tumor was suspected (Fig. 2).

**Fig. 3:**
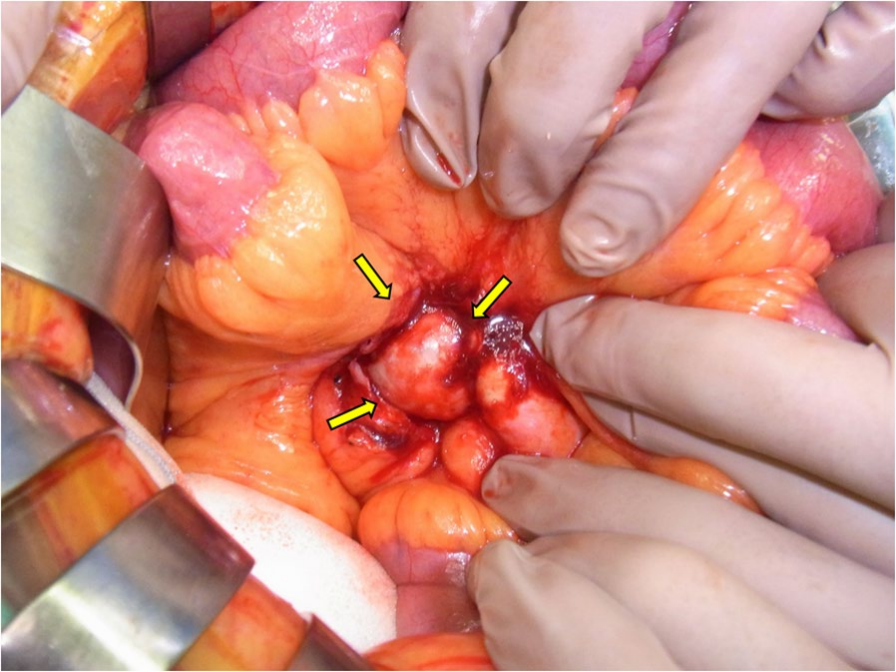
The tumor is located in the mesentery of the ileum and retracted into the mesentery (arrows)

**Fig. 4:**
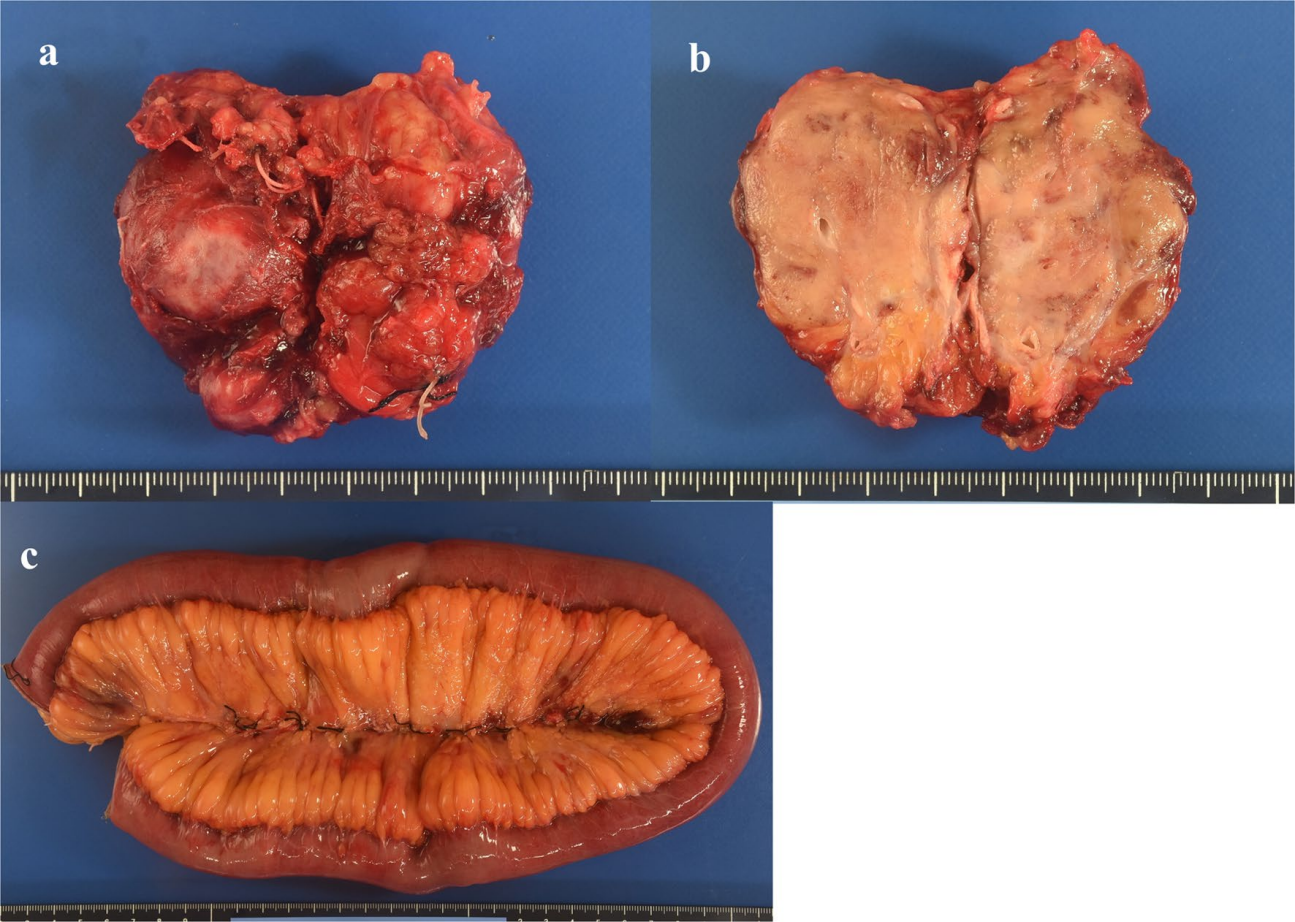
Resected specimens. **a** Tumor resected together with the surrounding mesentery. The tumor size is 55 × 33 × 33 mm^3^. **b** The cut surface is pale yellowish-white. **c** Additional resected ileum (90 cm). It is congested, but no tumor is found

Based on the imaging findings, we suspected a malignant mesenchymal tumor, and because the tumor was painful, surgery was performed to diagnose and treat it. The tumor was located in the mesentery of the ileum and retracted into the mesentery (Fig. 3). Surgery was performed via a laparotomy. Initially, the small mesenteric mass was resected along with the surrounding mesentery (Fig. 4a). However, resection of the tumor and mesentery resulted in impaired blood flow to the ileum in that area, and eventually, a 90-cm ileum was also resected (Fig. 4c). The cut surface of the 55 × 33 × 33 mm^3^ tumor was pale yellowish-white (Fig. 4b). Anastomotic reconstruction was performed using an instrumental functional end-to-end anastomosis. Our intraoperative search did not reveal any small bowel tumor, and an additional search of the resected ileum did not reveal any tumor. Thus, we diagnosed the patient with a primary ileal mesenteric tumor rather than a metastatic tumor.

**Fig. 5:**
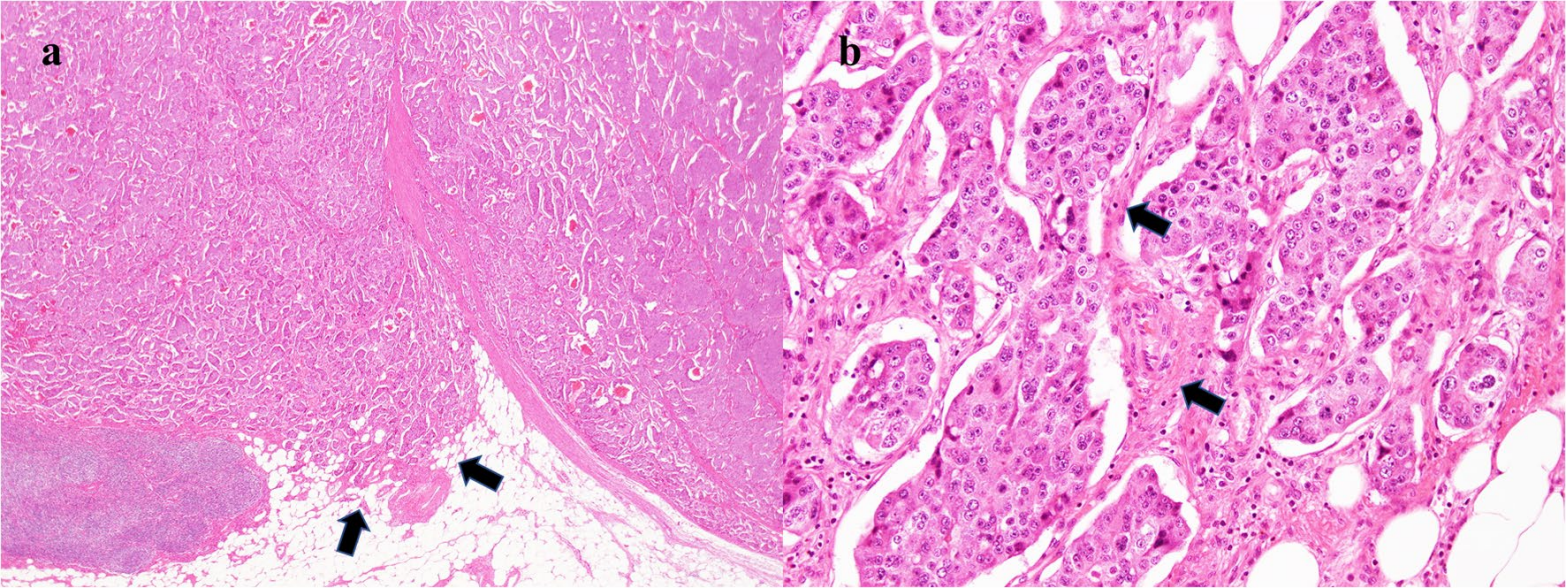
Histopathological findings (H&E). **a** (× 40) Proliferative infiltration of atypical cells of unequal size and irregular spore structure. Some of them infiltrate the surrounding adipose tissue (arrows). **b** (× 200) Proliferating atypical cells have round nuclei and relatively abundant cytoplasm with pale eosinophilia. There are narrow fibrovascular interstitial spaces between atypical cell foci (arrows)

**Fig. 6:**
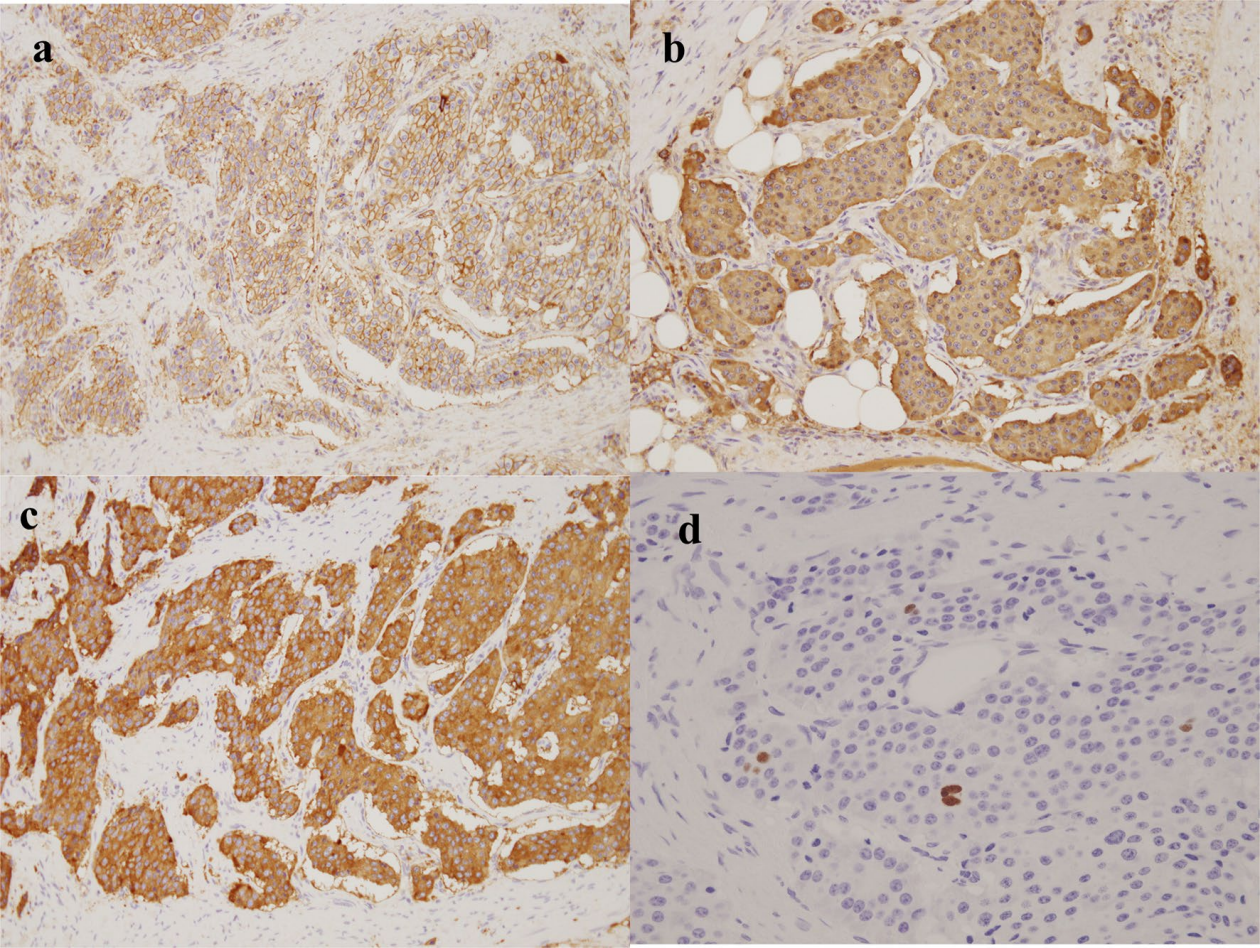
Immunohistochemical staining is positive for the following: **a** (× 200) CD56. **b** (× 200) chromogranin A. **c** (× 200) synaptophysin. **d** (× 400) The Ki-67 index is 3%

Histological examination showed proliferative infiltration of atypical cells of unequal size and irregular spore structure. Some atypical cells infiltrated the surrounding adipose tissues (Fig. 5a) and proliferating atypical cells had round nuclei and a relatively abundant cytoplasm with pale eosinophilia. There were narrow fibrovascular interstitial spaces between atypical cell foci (Fig. 5b). Immunohistochemical staining was positive for CD56 (Fig. 6a), chromogranin A (Fig. 6b), and synaptophysin (Fig. 6c). The Ki-67 index was 3% (Fig. 6d). The final pathological diagnosis was NET G2.

The patient’s postoperative course was good, and he was discharged 10 days after the surgery. The patient developed no recurrence for 1.5 years. Postoperative antitumor therapy was not performed for this patient because the histological diagnosis was NET G2, and it was determined that the tumor could be completely resected by surgery.

## Discussion

NETs are rare neoplasms. They arise from cells of the diffuse endocrine system, which are mainly dispersed throughout the gastrointestinal, pancreatic, and respiratory tracts. Neuroendocrine cells are derived from the epithelial and neuroectodermal cells. NETs are positive by silver staining and express synaptophysin, neuron-specific enolase, and chromogranin A.

The worldwide prevalence and incidence of NETs have recently increased. The incidence of NET was 1.09 per 100,000 people in 1973 and increased to 6.98 per 100,000 people in 2012 in the United States [2]. Based on data derived from the National Cancer Registry in Japan, the total number of patients treated for gastro-entero-pancreatic NENs (GEP-NENs) in 2016 was 6735, and the age-adjusted overall incidence was 3.53 per 100,000 people. The age-adjusted incidence of each primary NEN is presented in Table 1 [3]. Approximately half of the GEP-NENs involved the rectum (1.82 per 100,000 people), followed by the pancreas (0.697 per 100,000 people). The incidence of ileal NEN is 1% of the total GEP-NENs [3].

True primary solid tumors of the mesentery include NETs, fibromatoses, neurofibromas, teratomas, germ cell tumors, and primary neoplasms composed of smooth muscle, blood vessels, or fat [4]. Primary mesenteric NETs are very unusual, although secondary mesenteric involvement is common, reported as 40–80%, respectively [5]. We present an extremely rare case of an ileal mesenteric NET with benign or low-grade malignant characteristics on preoperative FDG-PET.

NETs are a subtype of NENs that are defined as epithelial neoplasms with predominant neuroendocrine differentiation. Because neuroendocrine cells are widely distributed throughout the body, NENs can arise in various locations, including the respiratory and digestive systems [6]. The WHO previously proposed a classification scheme for digestive NENs that divides them into three categories based on mitotic count and Ki-67 labeling index value: NET G1, NET G2, and NEC [7]. In particular, a mitotic count of < 2 per 10 high-power fields (HPFs) and/or Ki-67 index of < 3% corresponds to NET G1, a mitotic count of 2–20 per 10 HPFs and/or Ki-67 index of 3–20% corresponds to NET G2, and a mitotic count of > 20 per 10 HPFs and/or Ki-67 index of > 20% corresponds to NEC. In 2019, the WHO revised its former classification scheme and established a well-differentiated subtype of NET G3 from cases previously classified as NEC (Table 2) [8].

**Table 2:** Classification and grading criteria for NEN (2019) [8]

Terminology	Differentiation	Grade	Mitotic rate (mitoses/10 HPF)	Ki-67 index
NET G1	Well differentiated	Low	< 2	< 3%
NET G2		Intermediate	2–20	3–20%
NET G3		High	> 20	> 20%
NEC small-cell type (SCNEC)	Poorly differentiated	High	> 20	> 20%
NEC large-cell type (LCNEC)			> 20	> 20%
MiNEN	Well or poorly differentiated	Variable	Variable	Variable

The current gold standard for functional imaging of NETs is somatostatin receptor scintigraphy (SRS) with 111In-diethylenetriaminepentaacetic acid-octreotide [9]. Which is a proliferation marker reflecting the prognosis. FDG-PET is the most widely used nuclear medicine technique for functional imaging of cancer. However, FDG-PET has never been routinely used for imaging NETs, and its diagnostic performance remains unclear. The overall sensitivity rates of diagnostic imaging for NETs were reported to be 89% and 58% for SRS and FDG-PET, respectively [10]. FDG-PET is not sensitive in detecting low-grade primary gastrointestinal NETs. Tumor differentiation can be used to guide the selection of nuclear imaging modalities for staging of gastrointestinal and pancreatic NETs. SRS appears to be more sensitive than FDG-PET for well-differentiated NETs, whereas FDG-PET demonstrates superior sensitivity for poorly differentiated NETs [11]. The tumor presented by us was diagnosed as a benign or low-grade malignant tumor on preoperative FDG-PET. Based on the final pathological diagnosis, the tumor was diagnosed as NET G2. If SRS could have been performed preoperatively in our case, it may have been diagnosed as an NET with strong accumulation at the tumor site. When a neoplastic lesion of the small intestine or mesentery is diagnosed, it is possible that the tumor is an NET, but it is often difficult to confirm the tumor diagnosis preoperatively. However, if FDG-PET and SRS can be performed simultaneously at the time of tumor diagnosis, it may be possible to diagnose NETs, including GRADE.

Surgery is the mainstay treatment for localized gastrointestinal NETs, and may be curative in cases of R0 resection. To our knowledge, only 10 cases of primary small intestinal mesenteric NETs have been reported in the literature [5, 12–20] (Table 3). Of the 11 patients, including this patient, there were five men and six women, with a mean age of 61.3 (range, 48–74) years. Tumors were localized in the jejunal mesentery in six cases and in the ileal mesentery in five cases. As far as the grade classification of these 11 cases is known, there were no highly malignant G3 cases. Of these 11 surgical procedures, tumor resection was performed in five cases, and small bowel resection including the mesentery, was performed in six cases. In addition, one of the five tumor resections was laparoscopic. Regarding the surgical approach, we initially considered observing the lesion using laparoscopy. However, due to the irregular shape of the tumor and the fact that the tumor was invading the mesentery, we ultimately opted for laparotomy.

**Table 3:** Resected cases of primary small intestinal mesenteric NETs [5, 12–20]

No	Author	Year	Age/sex	Location	Size (mm)	Grade	Operation
1	Barnard	1984	74/M	Ileum	60 × 55	NA	Ileal resection
2	Stone	1993	48/F	Jejunum	40 × 32	NA	Tumor resection
3	Tsubaki	2003	73/F	Ileum	45 × 35	NA	Tumor resection
4	Yamanuha	2009	52/M	Ileum	20 × 20	NA	Ileal resection
5	Park	2013	73/F	Jejunum	82 × 73	G1	Tumor resection
6	Sakai	2013	56/F	Ileum	40 × 35	G1	Tumor resection
7	Jida	2014	59/M	Jejunum	77 × 77	G1	Jejunal resection
8	Ikenaga	2014	59/F	Jejunum	24 × 20	G1	Jejunal resection
9	Yasuda	2017	72/M	Jejunum	40 × 40	G1	Tumor resection
10	Tsuji	2019	56/F	Jejunum	16 × 16	G2	Laparoscopic tumor resection
11	Our case	2020	55/M	Ileum	55 × 33	G2	Ileal resection

*NA* not available

## Conclusions

Herein, we report an extremely rare case of primary ileal mesenteric NET resection. The preoperative diagnosis of a small intestinal mesenteric tumor is difficult. However, if FDG-PET shows a small accumulation in the tumor, the possibility of a well-differentiated NET G2 should be considered.

## Data Availability

Not applicable.

## Abbreviations

NEN: Neuroendocrine neoplasm
NET: Neuroendocrine tumor
NEC: Neuroendocrine carcinoma
WHO: World Health Organization
NET G2: Grade 2 neuroendocrine tumor
FDG-PET: Fluorodeoxyglucose-position emission tomography
CT: Computed tomography
GEP-NENs: Gastro-entero-pancreatic neuroendocrine neoplasms
HPF: High-power fields
SRS: Somatostatin receptor scintigraphy
